# Metformin and risk factors for chronic kidney disease in a European population based on Mendelian randomization

**DOI:** 10.1080/0886022X.2025.2486551

**Published:** 2025-04-28

**Authors:** Xiaopei Liu, Xingyao Li, Peng An, Qi Gao, Yanhong Zhao, Xingmin Shi, Xili Wu

**Affiliations:** a Department of Traditional Chinese Medicine, Huangling Hospital of Traditional Chinese Medicine, Yan’an, Shaanxi, China; b Department of Traditional Chinese Medicine, Second Affiliated Hospital of Xi’an Jiaotong University, Xi’an, Shaanxi, China; c Key Laboratory for Disease Prevention and Control and Health Promotion of Shaanxi Province, School of Public Health, Medical Science Center, Xi’an Jiaotong University, Xi’an, Shaanxi, China

**Keywords:** Mendelian randomization, metformin, chronic kidney disease, summary data-based Mendelian randomization, causal relationship

## Abstract

**Background:**

Metformin, widely used for type 2 diabetes, raises concerns about its use in chronic kidney disease (CKD) due to risks like lactic acidosis and renal function impact. This study uses Mendelian randomization (MR) and summary data-based MR (SMR) to explore metformin’s potential causal relationship with CKD and associated genes.

**Methods:**

We employed MR methods (MR-Egger, weighted median, IVW) and sensitivity analyses to explore the causal relationship between metformin and CKD. SMR was used to analyze eQTL and CKD data from the UK Biobank and FinnGen, intersecting these with metformin drug targets to identify genes associated with CKD.

**Results:**

MR analysis indicated that metformin may increase CKD risk (IVW model: OR = 144.67, *p* < 0.01). However, given the high OR value, additional studies are warranted to validate this finding. SMR identified genes ANPEP, STK11, ACACB, and RPS6KB as significantly associated with CKD risk.

**Conclusion:**

The study suggests metformin could elevate CKD risk and identifies relevant genes. Clinicians should exercise caution when prescribing metformin, particularly for patients with renal issues. Further research is needed to confirm these findings and guide clinical practices.

## 1. Introduction

As one of the most extensively used drugs for treating type 2 diabetes globally, metformin is widely recognized owing to its effective glycemic control and relatively good safety profile. However, its administration in patients with chronic kidney disease (CKD) remains significantly controversial [1–3]. CKD is featured by a progressive loss of kidney function, typically precipitated by diabetes, hypertension, and other factors [4]. Given the impaired renal function in patients with CKD, the metabolism and excretion of drugs may be impacted, increasing the risk of drug accumulation and relevant side effects. Especially concerning metformin, its association with lactic acidosis makes its administration more cautious and complex in patients with impaired renal ­function [5].

In recent years, with the in-depth study of the mechanism of metformin action and the management strategy of CKD, new perspectives on the application of metformin in CKD patients have gradually emerged in clinical practice. On the one hand, the traditional perspective focuses on avoiding the use of metformin in patients with impaired renal function to prevent severe metabolic complications. On the other hand, new studies and clinical guidelines suggest that metformin can still be safely and effectively employed in some CKD patients with proper monitoring and dose adjustment [6,7]. Such divergence not only reflects differing medical viewpoints on drug safety and efficacy but also underscores the importance of balancing risks and benefits in the management of chronic diseases.

Mendelian randomization (MR) uses genetic variation as an instrumental variable to assess the causal relationship, reduces confounding bias through random assignment of genes, effectively addresses the issues of reverse causal relationships, simulates randomized controlled trials, and provides robust causal inference. With the enrichment of genetic data, MR has been widely adopted in large-scale data studies, significantly enhancing the accuracy of causal relationship studies [8–10].

**Figure 1.**
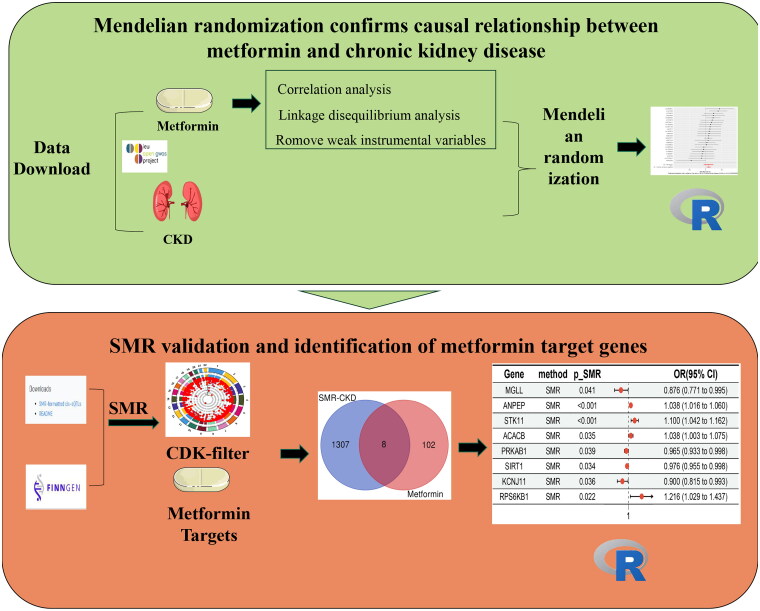
Flow chart.

To address the controversy over the application of metformin in CKD management, this study intends to expound the causal relationship between metformin and CKD with a two-sample MR method. The SMR approach was employed to integrate cis-eQTL and FinnGen data, prioritizing genes that may be pleiotropic or have potential causal relationships with the disease. Genes associated with CKD were screened out through the SMR analysis. Combined with the drug target genes of metformin, the study ultimately identified target genes and the causal relationship between metformin and CKD. The goal is to provide clinicians with more scientifically grounded guidance to better balance the drug risks and benefits in practice. The specific flow chart is shown in Figure 1.

## 2. Materials and methods

### 2.1. Data sources

#### 2.1.1. MR-data sources

The UKB-a-159 and finn-b-N14_CHRONKIDNEYDIS datasets were sourced from the Integrated Epidemiology Unit Open Genome-Wide Association Study (GWAS) database (https://gwas.mrcieu.ac.uk/) using the search terms ‘metformin, chronic kidney disease’. The UKB-a-159 dataset, related to metformin, comprises 337,159 samples and 10,894,596 single nucleotide polymorphism (SNPs). The finn-b-N14_CHRONKIDNEYDIS dataset includes 16,380,459 SNPs from 3902 CKD samples. In this study, metformin served as the exposure factor, and CKD was the outcome measure.

#### 2.1.2. SMR-data sources

The SMR cis-eQTL data were obtained from the eQTLGen consortium (version 20191212), which includes summary-level data from 37 datasets and 31,684 participants, available at https://www.eqtlgen.org/cis-eqtls.html. The GWAS data for chronic kidney disease were sourced from the R10 version of the FinnGen documentation [11], comprising 406,745 European participants (10,039 cases and 396,706 controls), accessible at https://finngen.gitbook.io/documentation/data-download.

#### 2.1.3. Metformin-data sources

The relevant data for metformin drug targets were obtained from TargetNet (http://targetnet.scbdd.com/calcnet/index/), DrugBank (https://go.drugbank.com/), SwissTargetPrediction (http://www.swisstargetprediction.ch/), DGIdb (https://www.dgidb.org/), and supplemented by literature.

#### 2.1.4. Three major assumptions of MR analysis

In MR analysis, there are three core assumptions: (1) a significant association between instrumental variables (IVs) and the exposure, (2) independence of IVs from confounders, and (3) IVs influence outcomes solely through exposure factors. These assumptions are critical for ensuring that causal inferences drawn from the analysis accurately reflect the true impact of exposure on disease or health outcomes.

##### 2.1.4.1. A robust and statistically significant correlation between IVs and exposure

IVs must be significantly associated with the exposure factor to ensure that it effectively represents the exposure. This constitutes the first major assumption of MR analysis. Typically, the relationship between IVs and the exposure is validated through statistical testing, with a commonly accepted threshold of a *p*-value < 5 × 10^−8^, indicating a strong association between IVs and exposure. To ensure the efficacy and significance of IVs, the extract instruments function was employed to extract IVs associated with the exposure from publicly available GWAS data.

SNPs with a *p*-value < 5 × 10^−8^ are generally selected as valid IVs. To minimize redundancy due to high linkage disequilibrium (LD), a clumping method was used to screen these SNPs. Independence between the selected IVs was ensured by setting the *R*^2^ threshold at 0.001 and the distance between SNPs (kb) at 10,000. The closer *R*^2^ is to 0, the lower the LD between IVs, promoting random allocation and ensuring that the independence assumption in the MR analysis is satisfied.

##### 2.1.4.2. Independence of IVs from potential confounders

The second key assumption in MR analysis is that there should be no systematic association between IVs and potential confounders. Confounders can distort the relationship between exposure and outcome, thereby compromising the accuracy of causal inferences. If an IV is correlated with a confounder, it undermines its validity as an IV, and the results of the analysis may incorrectly reflect the causal effect of exposure on the disease or outcome.

To ensure that IVs remain unaffected by potential confounders, their characteristics, and context are further verified through existing literature or databases. For instance, the extract_instruments function can be employed to assess the relationship between IVs and potential confounders. If any potential confounding effect is identified, those IVs may need to be excluded or replaced with more appropriate IVs.

##### 2.1.4.3. IVs affect outcomes only through exposure factors

The third assumption is that IVs influence outcome variables solely through exposure factors, not through other pathways or mediating effects. This assumption ensures that exposure is the only causal pathway, eliminating the possibility that IVs could impact the outcome through other non-exposure factors or pathways.

To validate this assumption, it is crucial to avoid selecting IVs that could affect the outcome through alternative pathways, such as mediating variables. The extract_outcome_data function can be used to retrieve IVs related to exposure factors from outcome data. In addition, strategies like setting proxy = TRUE can help address potential proxy effects.

##### 2.1.4.4. Analysis using the TwoSampleMR package

The TwoSampleMR package was employed for the analysis. The specific steps are as follows:

Screening for valid IVs: Exposure factors were evaluated using the extract_instruments function to select SNPs significantly associated with the exposure, typically those with a *p*-value < 5 × 10^−8^. LD analysis was performed by setting clump = TRUE to remove SNPs exhibiting correlation in LD, thereby ensuring independence among IVs. The selection was refined by setting *R*^2^ = 0.001 and kb = 10,000 to minimize correlation among the IVs.

Address the proxy effects: The extract_outcome_data function was used to retrieve the data of outcome variables and integrate it with the IVs associated with the exposure factors. The set of proxy = TRUE allows for the handling of potential proxy effects between the IVs and the outcome, thus avoiding the influence of incomplete or indirect measurements on the analysis results.

Exclusion of weak IVs: The validity of IVs was assessed using the *F*-statistic of each IV. Weak IVs can lead to assessment bias. Therefore, IVs with a low *F*-statistic were excluded. In general, an *F*-statistic >10 was considered necessary to ensure the potency of IVs to the exposure. Weak IVs attenuate the reliability of causal inferences and can lead to biased results. In addition, weak IVs were excluded based on the *F*-statistic for each SNP.

$$F=\frac{R^{2}}{1-R^{2}}\times \frac{N-K-1}{K}$$

In the formula, *R*^2^ indicates the total variance in the exposure explained by the selected instrument variables (IVs). *N* is the sample size of the genome-wide association study (GWAS) and *K* is the number of SNPs chosen for the exposure. An *F*-statistic ≥10 suggests that there is no weak instrument bias, confirming the IVs’ strong predictive power for the outcome.

### 2.2. MR

#### 2.2.1. MR analysis

Various MR methods were utilized to confirm the causal relationship between metformin and CKD, including IVW, MR-Egger, weighted median, simple mode, and weighted mode methods, with IVW being the primary method. A *p*-value < 0.05 using the IVW method was considered indicative of a potential causal relationship. Odds ratios (ORs) were calculated, where values >1 indicated risk factors and values <1 indicated protective factors. These ORs were used to create scatter plots, forest plots, and funnel plots for visual representation. Sensitivity analyses were then conducted using heterogeneity, horizontal pleiotropy, and leave-one-out (LOO) analysis to assess the reliability of MR results. In addition, heterogeneity tests were performed with *p* > 0.05 indicating no heterogeneity, and *p* > 0.05 in the horizontal pleiotropy test suggesting the absence of horizontal pleiotropy [12–14].

### 2.3. SMR analysis

The SMR analysis leveraged summary statistics from eQTL and GWAS datasets to investigate the association between gene expression and CKD. SNP markers from cis-eQTLs were employed as IVs, with gene expression serving as the exposure and CKD as the outcome. The analysis was conducted using SMR software version 1.3.1 with default settings. This included filtering SNPs with a minor allele frequency (MAF) >0.01, selecting cis-eQTLs with *p* < 5 × 10^−8^, excluding SNPs with linkage disequilibrium (LD) *R*^2^ >0.90 or <0.05 between top SNPs, and removing one SNP from each pair with LD *R*^2^ >0.90 (Zhu et al. 2016). Gene loci with P_SMR < 0.05 were identified as significant. The robustness of the associations was assessed using the heterogeneity in dependent instruments (HEIDI) test, with P_HEIDI >0.05 considered indicative of robust associations. The SMR analysis process is illustrated in Figure 1.

## 3. Results

### 3.1. MR

#### 3.1.1. Metformin has a causal relationship with CKD

**Figure 2.**
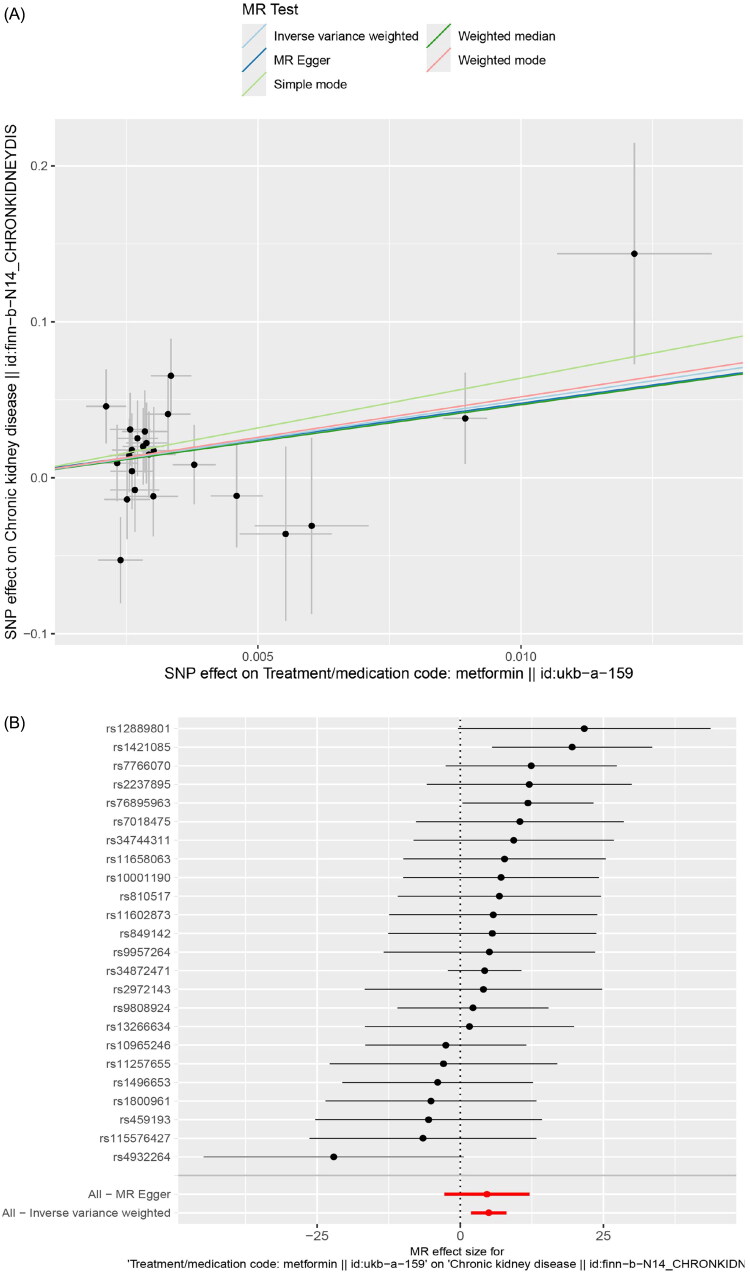

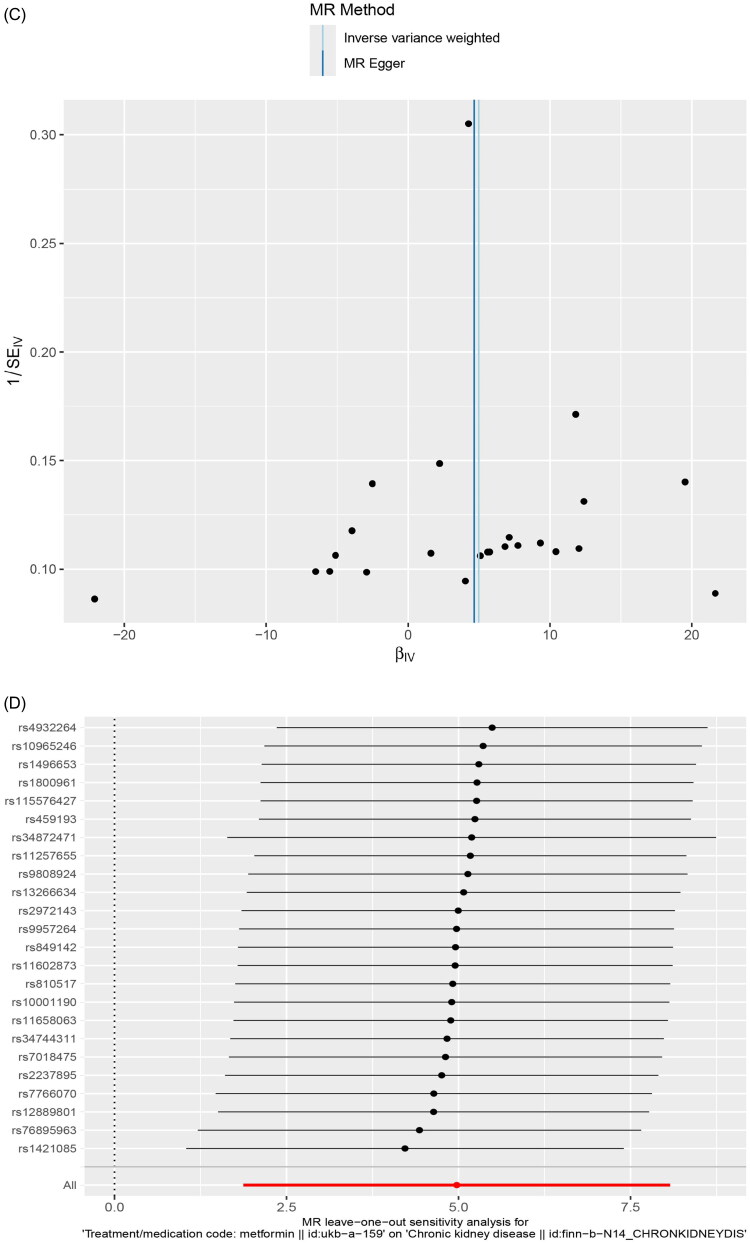
(A) Shows a scatter plot illustrating the causal relationship between metformin and CKD. SNP: single nucleotide polymorphism; (B) displays a forest plot of the single SNP effect on the causal relationship between metformin and CKD; (C) Figure 4 shows a funnel plot depicting the causal relationship between metformin and CKD. βiv: the β value of the instrumental variable; (D) presents a forest plot illustrating the leave-one-out analysis of the causal relationship between metformin and CKD.

After screening, 24 SNPs were selected as instrument variables (IVs) (Table 1). MR analysis was performed to evaluate the impact of metformin on chronic kidney disease (CKD) in the European population, with metformin as the exposure and CKD as the outcome. The IVW method in MR showed a significant positive causal relationship between metformin and CKD, indicating its role as a risk factor (IVW model: OR = 144.67, *p* = 0.002) (Table 2, Figure 2(A)). SNP scatter plots were generated to visually depict the relationship between the exposure and outcome. These plots demonstrated a clear positive linear trend, suggesting an association between higher levels of metformin exposure and increased risk of CKD development (Figure 2(A)).

**Table 1.** Detailed genome-wide association study (GWAS) data on exposure factors and outcomes.

	ukb-a-159	finn-b-N14_CHRONKIDNEYDIS
Year	2017	2021
Category	NA	Binary
Sub category	NA	NA
Population	European	European
Gender	Males and females	Males and females
Number of case group	8392	3902
Number of control group	328,767	212,841
Sample size	337,159	16,384,361
Number of SNPs	10,894,596	16,380,459

**Table 2.** Mendelian randomization analysis statistical data.

id.exposure	id.outcome	Method	nsnp	*p*val	or	or_lci95	or_uci95
ukb-a-159	finn-b-N14_CHRONKIDNEYDIS	MR Egger	24	0.23294061005476	105.242915536625	0.0617868132939579	179,262.70477737
ukb-a-159	finn-b-N14_CHRONKIDNEYDIS	Weighted median	24	0.0565961707908814	107.914343414771	0.876711214150215	13,283.1716153294
ukb-a-159	finn-b-N14_CHRONKIDNEYDIS	Inverse variance weighted	24	0.00166830012366477	144.67568695474	6.50807897924656	3216.1647795874
ukb-a-159	finn-b-N14_CHRONKIDNEYDIS	Simple mode	24	0.141059358201191	596.243553705734	0.160974473161566	2,208,464.28848896
ukb-a-159	finn-b-N14_CHRONKIDNEYDIS	Weighted mode	24	0.0802273680833557	179.227227662543	0.691901293511427	46,426.2741475427

IVW: inverse variance weighting; lci95: lower 95% confidence interval; MR: Mendelian randomization; nsnp: number of single nucleotide polymorphisms; OR: odds ratio; *p*val: *p*-value; uci95: upper 95% confidence interval.

To assess the predictive power of each SNP locus on the exposure and outcome, forest plots were created. In these plots, solid points on the left indicate lower risk, while those on the right indicate higher risk. Consistent forest plot results predominantly positioned solid points on the right, indicating that increased exposure to the factor, according to the IVW method, correlates with an increased risk of disease onset (Figure 2(B)). Concurrently, the randomness of instrument variables was evaluated and visualized using funnel plots. These plots showed a symmetrical distribution of IVs around the IVW line, confirming adherence to Mendelian randomization principles in the MR analysis (Figure 2(C)).

To ensure the robustness of our findings, we conducted comprehensive sensitivity analyses. Firstly, all *Q*_*p*val values from heterogeneity tests exceeded 0.05, indicating no significant heterogeneity between samples and highlighting the IVW (fixed effects) method as the primary approach in MR analysis (Table 3). Subsequently, MR-Egger and MR-PRESSO regression tests were performed to detect potential horizontal pleiotropic effects of genetic IVs. Consistent results across these tests showed no such effects between metformin and CKD (*p* > 0.05) (Tables 4 and 5). LOO analysis was employed to evaluate result reliability. This method systematically excluded one SNP at a time and performed MR with the remaining SNPs to assess the individual SNP’s significant impact on the outcome. The main goal of LOO analysis was to ensure smooth continuity of the connecting lines in the plot, without noticeable outliers. The results demonstrated no conspicuous outliers, affirming the reliability of the findings (Figure 2(D)).

**Table 3.** Cochran’s *Q* test to measure heterogeneity.

id.exposure	id.outcome	Method	*Q*	*Q*_*df*	*Q*_*p*val
ukb-a-159	finn-b-N14_CHRONKIDNEYDIS	MR Egger	22.1929155942289	22	0.44842555685443
ukb-a-159	finn-b-N14_CHRONKIDNEYDIS	Inverse variance weighted	22.2015112155784	23	0.50811594421539

IVW: inverse variance weighting; MR: Mendelian randomization; *Q*_*df*: degrees of freedom associated with Cochran’s *Q* test of heterogeneity; *Q*_*p*val: *p*-value of the *Q* test for heterogeneity test.

**Table 4.** MR-egger regression test to examine horizontal pleiotropic effects.

id.exposure	id.outcome	egger_intercept	*SE*	*p*val
ukb-a-159	finn-b-N14_CHRONKIDNEYDIS	0.0012208663645128	0.0132259029388601	0.927288174320583

*p*val: *p*-value; *SE*: standard error.

**Table 5.** MR-PRESSO_global detects the overall pleiotropy of all SNPs.

id.exposure	id.outcome	RSSobs	*p*val
ukb-a-159	finn-b-N14_CHRONKIDNEYDIS	23.8189489733259	0.543

*p*val: *p*-value; RSSobs: observed residual sum of squares.

### 3.2. SMR analysis

**Figure 3.**
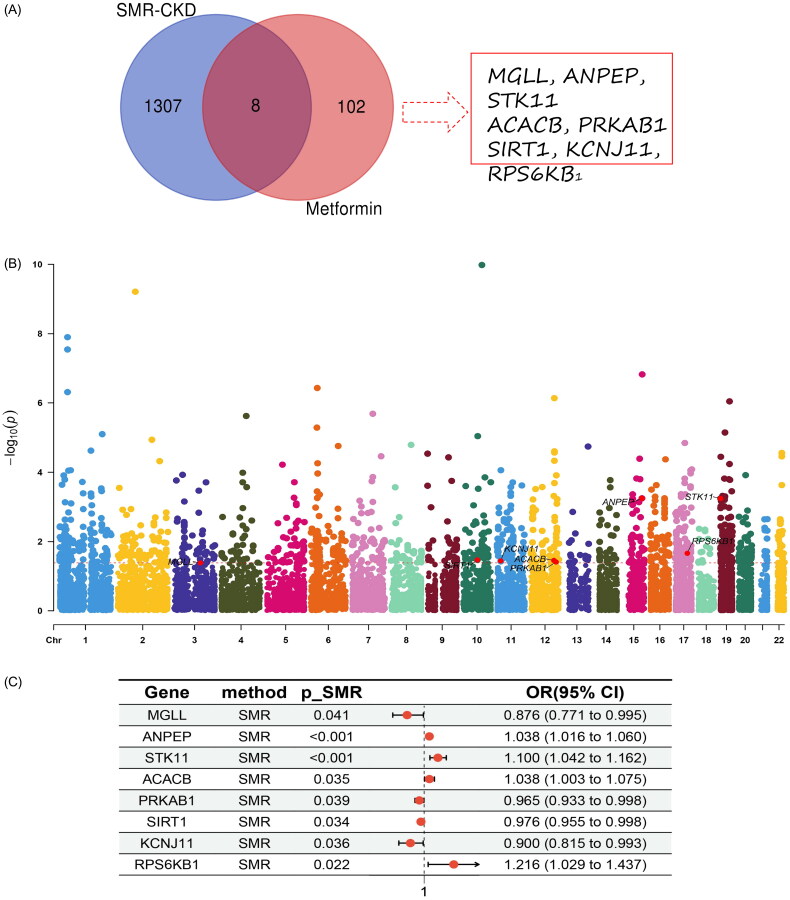
(A) Venn diagram of SMR-CKD and Metformin; (B) Manhattan plot of SMR analysis results for Metformin-targeted chronic kidney disease; (C) Forest plot of causal relationships between Metformin and chronic kidney disease proteins.

Through SMR analysis and subsequent filtering based on P_SMR and P_HEIDI values, 15,679 probes from the eQTLGen blood data were found to be associated with CKD. Among these, 1315 genes demonstrated pleiotropy or a potential causal relationship with CKD. After intersecting with metformin-related genes, eight genes were ultimately identified as having a causal relationship with CKD: MGLL, ANPEP, STK11, ACACB, PRKAB1, SIRT1, KCNJ11, and RPS6KB1. For detailed information on these genes, please refer to Figure 3 and Table 6.

**Table 6.** SMR genes significantly associated with metformin and CKD.

Ensemble ID	CHR	Gene	Top SNP	SMR beta	*p*-Value
ENSG00000074416	3	MGLL	rs664910	0.0193477	0.0394031
ENSG00000166825	15	ANPEP	rs11073891	−0.0275623	0.000558766
ENSG00000118046	19	STK11	rs3764640	−0.0303588	0.000513074
ENSG00000076555	12	ACACB	rs2268405	−0.0213059	0.034574
ENSG00000111725	12	PRKAB1	rs11064881	−0.0288054	0.0383743
ENSG00000096717	10	SIRT1	rs10997832	−0.0169594	0.0334981
ENSG00000187486	11	KCNJ11	rs2074310	−0.0166164	0.0332813
ENSG00000108443	17	RPS6KB1	rs1292060	0.0197511	0.0192066

## 4. Discussion

CKD is a public health problem worldwide, with an estimated global prevalence of 8–16%. In Western countries, ∼8–10% of the population is affected, while in the United States, over 15% of adults may have CKD [15,16]. In recent years, the administration of metformin in CKD patients has attracted considerable attention, given its potential risks and benefits [7,17].

### 4.1. Key findings

In this study, MR was used to determine a risk causal relationship between metformin and CKD, identifying metformin as a risk factor for CKD (IVW model: OR = 144.67, *p* < 0.01). SMR was employed to pinpoint the target genes associated with the risk of CKD onset in relation to metformin. The analysis revealed that ANPEP (IVW model: OR = 1.038, *p* < 0.001), STK11 (IVW model: OR = 1.1, *p* < 0.001, ACACB (IVW model: OR = 1.038, *p* = 0.035), and RPS6KB (IVW model: OR = 1.216, *p* = 0.022) may be related to the risk of CKD onset.

### 4.2. Mechanism of action of risk targets

The onset and development of CKD are closely linked to the abnormal expression and dysfunction of various genes. Our findings demonstrate that four genes, ANPEP, STK11, ACACB, and RPS6KB, may play a pivotal role in the progression of CKD. The potential mechanisms through which these genes may act as risk factors for CKD are as follows:

#### 4.2.1. Aminopeptidase N (ANPEP)

The ANPEP gene encodes aminopeptidase N, a zinc-dependent aminopeptidase located on the cell surface and widely distributed in tissues, such as the kidney, small intestine, and immune system. Inflammation and immune response: ANPEP modulates the processing of peptides and antigens, thereby engaging in the immune response. CKD patients are usually accompanied by chronic low-grade inflammation. ANPEP may affect glomerular and tubular function by regulating the inflammatory response, thus facilitating the progression of CKD [18,19]. Another study [20] has manifested that aberrant expression of ANPEP may be related to tubulointerstitial fibrosis, a common pathological feature of CKD. Exacerbated fibrosis leads to a progressive loss of kidney function.

#### 4.2.2. STK11 (serine/threonine kinase 11)

STK11 (also known as LKB1) is a tumor suppressor gene that encodes a protein critical for regulating cellular metabolism, polarity, and proliferation. Regulation of metabolism: STK11 is an upstream activator of AMP-activated protein kinase (AMPK), a key regulator of cellular energy balance. STK11 regulates renal metabolism and maintains cellular energy homeostasis by activating AMPK. Loss of function or mutation of STK11 may cause metabolic disorders and stress responses of renal tubular cells, contributing to the development of CKD [21]. STK11 also activates antioxidant defense mechanisms through the AMPK pathway, reducing the accumulation of reactive oxygen species and protecting kidney cells from oxidative stress. STK11 deficiency may augment the level of oxidative stress in kidney cells, thereby promoting the onset of CKD [22].

#### 4.2.3. ACACB (acetyl-CoA carboxylase beta)

As one of the key enzymes in fatty acid metabolism, the protein encoded by ACACB is responsible for converting acetyl-CoA to malonyl-CoA and is involved in lipid synthesis and oxidation. ACACB is crucial for lipid metabolism, regulating both the synthesis and oxidation of fatty acids. Disorders in lipid metabolism are strongly associated with the development of CKD, particularly in cases of obesity and kidney disease related to metabolic syndrome. The Aberrant expression of ACACB may induce renal lipid accumulation and metabolic dysregulation, further contributing to kidney damage. ACACB affects cellular energy balance by modulating the utilization and storage of fatty acids. Dysfunction of ACACB may result in insufficient energy supply to tubular cells and affect their normal function, thus accelerating the progression of CKD [23,24].

#### 4.2.4. RPS6KB (ribosomal protein S6 kinase B)

Members of RPS6KB family, including S6K1 and S6K2, belong to the serine/threonine protein kinase family and are involved in the regulation of protein synthesis, cell growth, and metabolism. As the significant downstream effector of the mTOR signaling pathway, RPS6KB is engaged in cell growth, protein synthesis, and metabolic regulation. Excessive activation of the mTOR signaling pathway is closely related to the progression of CKD, particularly in relation to glomerular hypertrophy and renal fibrosis. Aberrant activation of RPS6KB may accelerate these pathological processes. RPS6KB regulates ribosomal function and protein synthesis through the phosphorylation of S6 protein. CKD is often accompanied by the aberrant metabolism of protein. Aberrant expression of RPS6KB may affect the protein synthesis and function of kidney cells, facilitating CKD lesions [25].

In summary, the four genes ANPEP, STK11, ACACB, and RPS6KB play significant roles in the occurrence and progression of CKD by regulating inflammatory response, metabolic homeostasis, oxidative stress, and signaling pathways. Aberrant expression or functional defects in these genes may contribute to the development of CKD through various mechanisms. Therefore, studying these genes is of great significance for understanding the pathological mechanism of CKD and developing potential therapeutic targets.

### 4.3. Possible explanation for metformin as a risk factor of CKD

#### 4.3.1. Pharmacokinetic effects

Metformin is predominantly excreted through the kidneys. Therefore, the clearance of metformin is significantly reduced in patients with renal insufficiency, leading to its accumulation in the body. Dosage adjustments for metformin are generally recommended for patients with mild to moderate renal impairment. However, for patients with severe renal insufficiency (eGFR < 30 mL/min/1.73 m^2^), metformin is traditionally contraindicated [26].

#### 4.3.2. Metformin and lactic acidosis

Despite its low incidence, lactic acidosis is one of the most severe potential side effects (∼0.03 cases/1000 patients per year) in metformin users. Lactic acidosis is mainly characterized by elevated serum lactate levels (typically >5 mmol/L) and decreased serum pH, accompanied by symptoms, such as fatigue, muscle pain, shortness of breath, and abdominal pain [27,28]. In CKD patients, renal function impairment may exacerbate the burden of metformin metabolism, inducing further deterioration of renal function. Due to the attenuated ability of the kidneys to clear metformin, the drug tends to accumulate in the body, elevating the risk of lactic acidosis [29,30]. In addition, CKD patients often present with other comorbidities (for instance: heart failure, infection) that may also increase the risk of lactic ­acidosis [31].

#### 4.3.3. Instructions for use of metformin in CKD patients

For managing the risk of lactic acidosis, the traditional perspective holds that patients with renal insufficiency should avoid using metformin. However, for patients with mild to moderate renal insufficiency (eGFR 30–60 mL/min/1.73 m^2^), it is recommended to adjust the dose of metformin based on renal function. For instance, patients with an eGFR of 45–60 mL/min/1.73 m^2^ may use the standard dose, while those with an eGFR of 30–45 mL/min/1.73 m^2^ require a dose reduction. Metformin is generally not recommended for patients with severe renal insufficiency (eGFR < 30 mL/min/1.73 m^2^). CKD patients should undergo regular renal function assessment (such as eGFR measurements) to ensure the safe use of metformin [32–35].

### 4.4. Clinical significance

#### 4.4.1. Optimize treatment strategies of diabetes

Individualized treatment regimen: studying the risk factors for metformin in CKD patients can help establish an individualized treatment regimen. By having a detailed understanding of each patient’s renal function status, healthcare providers can properly adjust the metformin dose or choose alternative drugs, thus avoiding drug accumulation in the body and reducing the risk of lactic acidosis.

Option of medication combination: studies have demonstrated that some drugs can provide better glycemic control and renal protection when combined with metformin. For instance, SGLT2 inhibitors and GLP-1 receptor agonists exert cardiovascular and renal protective effects in CKD patients [36–39]. By studying the risks of metformin, clinicians can be better guided in choosing the optimal medication combination strategy.

#### 4.4.2. Improve patient safety

Monitoring and prevention of lactic acidosis: studying the potential risks of metformin can enhance vigilance for lactic acidosis. Clinicians can develop a rigorous monitoring plan to regularly assess the patient’s renal function and lactate levels, ensuring timely detection and prevention of the occurrence of lactic acidosis.

Patient education and management: understanding the risk factors for metformin can help the medical team better educate patients about the potential side effects and early symptoms of the drug. Therefore, patients can quickly identify and seek medical attention when they experience symptoms, such as fatigue, shortness of breath, and muscle pain, thereby reducing the incidence of serious complications.

#### 4.4.3. Promote clinical guideline updates

Data-driven guideline updates: as research progresses, new data and evidence will help update clinical guidelines. Existing guidelines may be conservative in some respects. By investigating the specific risks and safety boundaries of metformin, more accurate and practical clinical guidelines can be developed to guide the safe use of metformin in CKD patients.

Improve the applicability and flexibility of guidelines: studies can contribute more data on the use of metformin in patients exhibiting varying degrees of renal insufficiency, making clinical guidelines more applicable and flexible. For example, in patients with mild and moderate renal insufficiency, complete discontinuation of metformin may be unnecessary. Instead, individualized dose adjustments can be considered on a case-by-case basis.

#### 4.4.4. Improve long-term health outcomes

Reduce the incidence of complications: glycemic control in CKD patients can be improved with the appropriate use of metformin, thereby decreasing the incidence of diabetes-related complications (for example: retinopathy, neuropathy, and cardiovascular disease) [40]. Delay the deterioration of renal function: proper hypoglycemic therapy can slow the progression of CKD. A study [41] has shown that metformin not only effectively controls blood glucose at appropriate doses, but may also confer renal protective effects. By studying its risks and benefits in CKD, optimizing medication regimens becomes feasible, thereby delaying the progression of renal function deterioration.

#### 4.4.5. Economic and social benefits

Reduce medical costs: effective management of metformin administration in CKD patients can mitigate emergency treatments and hospitalizations associated with lactic acidosis, thus easing the burden on the healthcare system. Meanwhile, optimizing diabetes management and reducing the occurrence of complications can lead to substantial reductions in long-term medical expenses. Improve patient’s quality of life: by employing metformin and other hypoglycemic medications judiciously, CKD patients can maintain good glycemic control, decrease complications, and enhance their overall quality of life. This is not only beneficial to patients, but also alleviates the care burden on the family and society.

#### 4.4.6. Facilitate interdisciplinary cooperation

Exploring the risk and management strategies of metformin in CKD patients requires multidisciplinary collaboration across fields, such as endocrinology, nephrology, pharmacology, and public health. With interdisciplinary studies, the risks and benefits of metformin can be comprehensively assessed, paving the way for the development of more scientifically sound and effective clinical practice guidelines.

### 4.5. Advantages and limitations

#### 4.5.1. Advantages

Most previous studies on the relevance between metformin use and adverse events are observational studies. However, due to factors, such as confounding variables and inadequate study design, the analysis of such observational data may generate erroneous associations. The association effectiveness between metformin use and adverse events is particularly worrisome, as metformin is frequently prescribed to patients with multiple comorbidities, which themselves may be the true causes of adverse outcomes. This is commonly referred to as confounding.

In this study, MR was used to effectively reduce the interference of confounding factors, such as social environment and lifestyle, as well as the impacts of reverse causal relationships on the results, whereas the sensitivity analysis further improved the reliability and stability of the results. The study utilized data from a less heterogeneous European ancestry population. Genome-wide association study (GWAS) data were sourced from two independent samples with relatively large sample sizes and the maximum statistical power.

## 5. Limitations and prospects

1. Firstly, the two-sample MR used in this study mainly addresses the linear association between exposure and outcomes. However, it’s unable to reveal potential nonlinear relationships. In biomedical research, the association between many exposures and disease outcomes is often more complex than a simple linear relationship. For instance, the effect of metformin on CKD may exhibit nonlinear effects across different dose ranges or treatment duration. This complex relationship cannot be captured by traditional linear models. Therefore, reliance solely on two-sample MR may not comprehensively assess the true effect of metformin on CKD, particularly when factors, such as dose response, treatment duration, or other unmeasured factors influence the effect. In addition, the study only included data from the European population that was sourced from European GWAS resources. This limitation restricts the external validity of the findings, particularly in terms of their applications on a global scale. European populations exhibit specific genetic backgrounds, lifestyles, and environmental factors, which may differ from those of other regions and ethnic groups. As a result, the effect of metformin on CKD could vary significantly across other ethnic groups. For instance, differences in metabolic processes, drug-metabolizing enzyme activity, and kidney function may exist across Asian, African, or Latin American populations. These differences could lead to divergent effects of metformin in these groups compared to European populations. Therefore, the generalizability of the findings from this study to different ethnic and geographical backgrounds remains unverified. Further research is needed to confirm the effect of metformin on CKD in other populations. Future studies should expand the sample size to include different ethnic groups and regions, providing a more comprehensive assessment of the impact of metformin on CKD on a global scale. Meanwhile, incorporating nonlinear analyses and including a more diverse set of populations will enhance our understanding of the mechanisms of metformin’s action and its differential efficacy across various groups.

2. Secondly, the absence of GWAS data stratified by sex and age significantly limits our ability to evaluate whether the association between metformin and CKD varies across different population groups. Gender and age are important biological factors that can influence drug metabolism, mechanisms of action, and the onset and progression of diseases. Without stratified analysis, it’s impossible to fully uncover the potential differential effects of metformin on male and female populations, as well as on younger and older individuals. This limitation not only hinders our ability to fully understand the impact of metformin on CKD risk but also restricts our capacity to deeply interpret its underlying biological mechanisms. For instance, the pharmacokinetic properties of metformin may vary across different groups based on sex and age. In certain situations, women may be more susceptible to the drug’s effects than men. In older populations, diminished kidney function could affect the rate of drug clearance, inducing different pharmacological responses [30,42]. In addition, the pathogenesis and progression of kidney disease may differ among patients of varying ages, especially in older individuals. CKD in this group is often accompanied by other chronic diseases and polypharmacy, which can significantly affect the efficacy of metformin [43,44]. Therefore, the lack of gender and age-stratified data prevents a deeper exploration of these potential group differences and affects the personalized treatment potential of metformin across different populations. Future studies incorporating stratified analyses based on sex and age will enable a more precise evaluation of metformin’s impact across different groups, contributing to elucidating its underlying biological mechanisms and providing significant basis for developing personalized treatment strategies.

3. The higher OR value (144.67) warrants further scrutiny of the results. In this study, IVs were meticulously validated to ensure that the associations between the selected IVs and exposure factors were both strong and significant. Known confounders were controlled using rigorous statistical methods to minimize the impacts of bias. However, extremely high OR values may still suggest the presence of unidentified confounders or other potential sources of bias that require additional investigation. For instance, there may be factors not fully accounted for in the current analysis that, although excluded, could still affect the association between exposure and outcomes to some degree. These potential confounders may include genetic background, lifestyle, environmental factors, or other unmeasured biomarkers that were not adequately controlled in the current dataset. In addition, the potential association between IVs and these unidentified confounders could contribute to the high OR value. Another possible source of bias may stem from weak IVs. While the valid IVs were screened through *F*-statistics and LD analysis, it remains possible that some IVs may not fully satisfy all assumptions, potentially leading to bias in the estimates. Weak IVs may fail to completely eliminate potential confounding effects, thus compromising the accuracy of causal inferences. Therefore, despite the effort made in the study to control known confounding factors, further validation is required to verify the existence and impact of these potential biases. Future studies should aim to confirm these findings and ensure the reliability of causal inferences by incorporating additional variable controls, utilizing stronger IVs, and conducting more extensive sensitivity analyses.

## 6. Conclusion

Studies employing MR have indicated that metformin use can increase the risk of CKD as an exposure factor. Clinicians should exercise caution regarding long-term metformin use, especially in individuals who are already at high risk of renal insufficiency in CKD. However, given the extremely high OR value and the limitations of the available data, further studies are necessary to validate these conclusions. In particular, repeated validation across different populations and under varying conditions is needed to ensure the generalizability and robustness of the study results. However, the effects and underlying mechanisms of metformin on CKD remain obscure. The research group will prioritize long-term prospective studies for future research in this area to establish a clearer causal relationship between metformin use and CKD outcomes. These studies should encompass various populations and explore changes in metformin dosage and treatment duration. In addition, there is a critical need for mechanistic studies to elucidate how metformin affects CKD at the molecular and cellular levels, potentially generating new prevention strategies or alternative treatments. The research group will incorporate real-world data to assess the impact of metformin in various clinical scenarios, conferring a more comprehensive understanding of its effects on CKD.

## Data Availability

The original contributions presented in the study are included in the article, further inquiries can be directed to the corresponding author.
